# Dialkyl Succinates and Adipates as Alternative Plasticizers—Even More Efficient Synthesis

**DOI:** 10.3390/ma14206219

**Published:** 2021-10-19

**Authors:** Natalia Barteczko, Justyna Więcławik, Anna Tracz, Ewa Pankalla, Karol Erfurt, Piotr Latos, Sławomir Boncel, Karolina Matuszek, Anna Chrobok

**Affiliations:** 1 Department of Chemical Organic Technology and Petrochemistry, Silesian University of Technology, Krzywoustego 4, 44-100 Gliwice, Poland; Natalia.Barteczko@polsl.pl (N.B.); Justyna.Wieclawik@polsl.pl (J.W.); Karol.Erfurt@polsl.pl (K.E.); Piotr.Latos@polsl.pl (P.L.); 2 Grupa Azoty Zakłady Azotowe Kędzierzyn, S.A., Mostowa 30A, 47-220 Kędzierzyn-Koźle, Poland; Anna.Tracz@grupaazoty.pl (A.T.); Ewa.Pankalla@grupaazoty.pl (E.P.); 3 Department of Organic Chemistry, Bioorganic Chemistry and Biotechnology, Faculty of Chemistry, Silesian University of Technology, Krzywoustego 4, 44-100 Gliwice, Poland; 4 School of Chemistry, Monash University, Clayton, VIC 3800, Australia; Karolina.Matuszek@monash.edu

**Keywords:** plasticizers, succinate esters, adipates esters, ionic liquids, acidic catalysis, esterification, solvents

## Abstract

As a result of strict regulations of phthalate plasticizers, alternative non-phthalate forms are desired and increasingly used. This work presents a synthetic method for alternative plasticizers (dialkyl succinates and adipates) via esterification of succinic and adipic acid with alcohols: butan-1-ol and 2-ethylhexan-1-ol. Ionic liquids were synthesized by the reaction of triethylamine with over-equimolar (1:2.7) amounts of sulfuric(VI) acid, which were used as an acidic catalyst and solvent. The two-phase liquid–liquid system was formed during the reaction due to immiscibility of the esters with the ionic liquid. This phenomenon is a driving force of this process, shifting the equilibrium toward the product formation. As a result, dialkyl succinates and adipates were obtained in high yields (99%) and selectivities (>99%), under mild reaction conditions at 70–80 °C and using a 4:1 molar ratio of alcohol to acid and 15 mol% of catalyst. The catalyst was recycled 10 times without any loss of activity. This alternative method is highly competitive: it involves a simple procedure for product isolation as well as a high yield and purity of the resulting esters. These advantages make this method sustainable and promising for industrial applications.

## 1. Introduction

Until recently, the most popular and produced plasticizers were phthalates, in particular bis (2-ethylhexyl) phthalate, di-*n*-butyl phthalate, di(isononyl) phthalate, di(isodecyl) phthalate and di-*n*-octyl phthalate. In 2015, the European Union introduced legal regulations that limited the use of phthalates due to their toxicity. Similar regulations limiting the use of phthalates are respected in North America, while in South America, India and China their use is not currently regulated by legal standards [1].

Many alternative non-phthalate plasticizers are now used including alkyl esters such as: terephthalates, adipates, succinates, trimellitates, benzoates, phosphates, sebacates, citrates, maleates, fumarates and epoxidized vegetable oils (soybean, rapeseed) [2,3,4]. Among them, the most popular are dialkyl succinates such as dioctyl succinate (DOS), dihexyl succinate, dibutyl succinate (DBS) and diethyl succinate. Succinate-based plasticizers (80–90% of world production) are most often used for poly(vinyl chloride) (PVC), which is considered to be the most plasticized polymer, as well as for other polymers: polyacrylates, polyvinyl acetate, polyolefins, polyurethanes and natural rubber [5,6,7]. Dialkyl succinates with longer alkyl chains, e.g., DOS, are the most effective plasticizers with similar or even better plasticizing properties than phthalate esters, having a high compatibility with the polymers that allows a high plasticization efficiency. Succinates with shorter alkyl chains achieve similar plasticizing properties to phthalates but require higher concentrations. Alkyl succinates originate from biomass conversion, are stable, biodegradable and are very often used in combination with epoxidized soybean oil (ESO). DOS is used in a wide variety of products including cosmetics and personal care products, adhesives and sealants, coating products, inks and toners and lubricants. It is also used as an additive to detergents, in car wash and care liquids, paints, fragrances and air fresheners [8,9].

Alcohols with the same alkyl chain length as phthalate esters can be also esterified with adipic acid to obtain adipate plasticizers, e.g., esterification of 2-ethylhexan-1-ol with adipic acid gives bis (2-ethylhexyl) adipate (DEHA). Adipic acid esters are widely used as PVC plasticizers due to their good performance at low temperatures and their lower viscosity than phthalate plasticizers. The most frequently used adipates have chain lengths up to C10, with longer chains becoming physicochemically incompatible with many polymers [10]. Additionally, adipates show greater variability and a higher degree of migration than phthalates and are more expensive due to their specialized use. The group of adipate plasticizers is characterized by a significant improvement in the flexibility of the material at low temperatures, which is due to the linear structure of the molecule and results in a lower viscosity of adipates as compared to phthalates and other plasticizers [11,12]. Moreover, Hocevar et al. recently reported that adipic acid can be directly produced from bio-based sources [13]. They presented the first heterogeneous catalytic process for converting an aldaric acid into muconic and adipic acid.

The non-phthalate plasticizers described above are produced via an esterification reaction which is carried out in the presence of acidic catalysts at high temperatures (above 100 °C), which can cause undesirable degradation reactions of alcohols to olefins or ethers. Additionally, the water–alcohol azeotrope needs to be distilled off to shift the equilibrium towards the product formation at a temperature of about 185 °C. An important issue, which is an object of extensive investigation in the esterification processes, is to manipulate the equilibrium of the reaction. An excess of the cheaper reagent or methods for the effective product isolation from the reaction mixture are the most often-used tools [14].

For example, di-*n*-butyl adipate was synthesised in the presence of MOFs (1 mol%) as a catalyst with a 99.9% yield after 60 min at 190 °C, using a 10-fold excess of alcohol in relation to the acid [15].

Di-*n*-butyl succinate was obtained with 91.6% yield in the presence of Lewis acid sites of Mg^2+^-modified polystyrene sulfonic acid resin at 120 °C after 60 min, using a 3-fold excess of 2-ethylhexan-1-ol to succinic acid [16].

Di(2-ethylhexyl)adipate can be obtained in an enzymatic esterification process catalysed by Candida Antarctica lipase B. This method allows for the achievement of almost 100% yield of the product when the process is carried out at a temperature of 50 °C for 180 min under a reduced pressure of 6.7 kPa [17].

The synthesis of di(2-ethylhexyl) succinate in the presence of a heterogeneous nano-SO_4_^2−^/TiO_2_ catalyst was also described. The product, with a 97% yield, was obtained at 160 °C after 120 min of rection [18].

Another example of acidic catalyst activated with bentonite was used for the synthesis of di-*n*-butyl succinate using 3.5-fold excess of alcohol at 130 °C. Under these conditions, the product was obtained after 180 min with 98.5% yield [19].

However, these methods are expensive and energy-consuming. A more novel approach adopts acidic ionic liquids (ILs) as catalysts and solvents. In this system, reagents and water are dissolved in the ionic liquid phase and the ester forms the second liquid phase. A continuous removal of the ester to the second phase shifts the equilibrium towards the ester formation [20,21].

Lewis acidic ILs obtained in the reaction of 1-butylpyridinium chloride and aluminium chloride were used for the initial esterification studies [22]. Due to the low levels of stability of Lewis ILs when in the presence of moisture, they cannot be applied for this process. On the other hand, Brønsted acidic ILs are stable in the presence of water and they have been successfully used in esterification reactions. Brønsted acidic functionality in ILs can be introduced to cation, anion, or both, bearing: (a) a proton on the quaternary nitrogen atom of the cation; (b) a protonated acidic group on the cation alkyl chain (–SO_3_H); (c) an available proton on the anion ([HSO_4_]^−^) [23,24,25,26]. Simple synthesis of Brønsted acidic ILs, based on the proton transfer between strong inorganic acids (H_2_SO_4_, HNO_3_, CF_3_COOH) and an *N*-base such as imidazole, pyrrolidine, piperidine or trialkylamine, makes this process not only effective but also economically viable [27]. Moreover, the structure of Brønsted acidic ILs can be tuned to find the proper acidity of the catalyst. For example, sulphuric(VI) acid can be used in various molar ratios, *χ*_H2SO4_ = 0.50, 0.67 or 0.75 in regard to the base. In our previous work, we demonstrated that the Gutmann acceptor number (AN) value measured for protic ILs based on 1-methylimidazole, for *χ*_H2SO4_ = 0.50 ([Hmim] [(HSO_4_)]), showing rather medium acidity (AN = 73.3). In this case, the acidity arises from the presence of the labile proton at the cation as well as the hydrogensulfate anion [28], whereas ILs obtained with an excess of H_2_SO_4_ (*χ*_H2SO4_ = 0.67 or 0.75) exhibit high acidity expressed by AN = 117–122 [28], similar to the acidity of strong acids such as CF_3_COOH (AN = 105.5) and CF_3_SO_3_H (AN = 129.1) [29]. Synthesis of these more acidic ILs leads to the formation of a hydrogen-bonded network of H_2_SO_4_ molecules and [HSO_4_]^−^ anions in an anion structure. The resulting ILs were used as both the acidic catalysts and as the solvent for the esterification of levulinic, terephthalic and lactic acids [30,31,32], as well as for the synthesis of ε-caprolactam [33].

Herein, we present the efficient method for the synthesis of dialkyl (di-*n*-butyl and dioctyl) succinates and adipates in the presence of low-cost Brønsted acidic ILs. This work contributes to the development of sustainable and environmentally-friendly processes for the production of green plasticizers.

## 2. Materials and Methods

### 2.1. Materials

Triethylamine (>99%), sulphuric(VI) acid (95%) and *n*-decane (>99%) were purchased from Sigma-Aldrich (Merck, Darmstadt, Germany). Adipic acid was purchased from BASF SE (Ludwigshafen am Rhein, Germany). Succinic acid (99%) was purchased form Acros Organic (Thermo Fisher Scientific, Waltham, MA, USA). Butan-1-ol and 2-ethylhexan-1-ol were produced at the plants of Grupa Azoty Zakłady Azotowe Kędzierzyn S.A. Deuterated solvents, e.g., dimethyl sulfoxide-d6 (99.8%) and chloroform-d (99.8%), were purchased from Sigma-Aldrich (Merck, Darmstadt, Germany).

### 2.2. Methods

GC analyses were performed on a Shimadzu GC-2010 Plus (Kyoto, Japan) (carrier gas–helium) with an oven temperature program: start at 80 °C isothermal for 1 min, then reach 300 °C with a heating rate of 40 °C/min and then 5 min at 300 °C isothermal. The Shimadzu GC-2010 Plus was equipped with a flame ionization detector (FID) and a Zebron ZB-5MSi column (30 m × 0.25 mm 0.25 μm film).

The NMR spectra of the products were recorded using a Varian 400 spectrometer (Palo Alto, CA, USA.) at the following operating frequencies: ^1^H 400 MHz and ^13^C 100 MHz. Chemical shifts are reported as parts per million (ppm) in reference to tetramethylsilane (TMS) for 0.020 g of the sample dissolved in 0.5 mL of deuterated solvent, e.g., DMSO-d6 or CDCl_3_.

### 2.3. Synthesis

Ionic liquids: three ILs with different molar ratios of amine to H_2_SO_4_ (1:3, 1:2.7 and 1:2) were prepared, later called IL 3.0, IL 2.7 and IL 2.0, respectively. Triethylamine (25.30 g, 0.25 mol) and the water solution (water 10 wt.% in regard to acid and base) of sulphuric(VI) acid (IL 3.0: 74.2 g; IL 2.7: 66.8 g; IL 2.0: 49.5 g) were slowly added dropwise to the batch jacket reactor (250 mL) equipped with a thermometer, a condenser and a mechanical stirrer, keeping the temperature at 40 °C (7 Hz, 20 min). Next, the reaction mixture was stirred for 60 min at 25 °C. Subsequently, water was evaporated and ILs were dried on high vacuum (80 °C, 10^−^^2^ mbar, 720 min). The obtained ILs were colourless liquids with a 98% yield. ^1^H NMR (400 MHz, DMSO) δ 8.95 (s, 1H), 7.51 (bs, 5H), 3.07 (q, 6H, J = 6.4 Hz), 1.15 (t, 9H, J = 7.9 Hz). ^13^C NMR (100 MHz, DMSO) δ 48.02, 12.20.

Succinate and adipate esters: IL 2.7 (0.75–0.92 g, 2.10–2.50 mmol, 15 mol%), alcohol (10.69–13.23 g, 0.08–0.10 mol) and acid (2.00 g, 0.014–0.02 mol, molar ratio of acid to alcohol: 1:4) were mixed at 70 °C for 240 min in a round-bottom reactor (25 mL), equipped with a reflux condenser, a temperature probe, an oil bath and a magnetic stirrer. At the end of the process, the phases were separated. Hexane (20 mL) was added to the upper phase, which was then extracted with water (3 × 20 mL), washed with a saturated aqueous solution of NaHCO_3_ (10 mL) to remove the traces of acidic IL and, finally, concentrated using a rotary evaporator. Next the alcohol was removed by vacuum distillation at 63–65 °C (10 mmHg) for 2-ethylhexan-1-ol and 115–118 °C (760 mmHg) for buthan-1-ol.

2-ethylhexyl succinate was obtained in 82% yield (5.68 g) as a colourless liquid; ^1^H NMR (400 MHz, CDCl_3_) δ 4.01 (m, 4H), 2.63 (s, 4H), 1.55 (m, 2H), 1.32 (m, 12H), 0.89 (m, 12H).

Di-*n*-butyl succinate was obtained in 83% yield (3.98 g) as a colourless liquid. ^1^H NMR (400 MHz, CDCl_3_) δ 4.09 (t, 4H, J = 6.69 Hz), 2.62 (s, 4H), 1.61 (dt, 4H, J_1_ = 6.77 Hz, J_2_ = 14.66 Hz), 1.38 (m, 4H), 0.93 (t, 6H, J = 7.38 Hz).

2-Ethylhexyl adipate was obtained in 91% yield (4.93 g) of as a colourless liquid. ^1^H NMR (400MHz, CDCl_3_) δ 3.99 (m, 4H), 2.33 (s, 4H), 1.67 (m, 4H), 1.56 (m, 2H), 1.53 (m, 4H), 1.33 (m, 12H), 0.89 (m, 12H).

Di-*n*-butyl adipate was obtained in 89% yield (3.56 g) as a colourless liquid. ^1^H NMR (400MHz, CDCl_3_) δ 4.05 (m, 4H), 2.33 (m, 4H), 1.59 (m, 8H), 1.35 (m, 4H), 0.89 (m, 6H).

### 2.4. Esterification Procedure

To monitor the progress of esterification reactions, a series of experiments was performed. The experiments were carried out in a round-bottom reactor (25 mL) equipped with a reflux condenser, a temperature probe, an oil bath and a magnetic stirrer. Depending on the experiment, the appropriate amounts of acid (2.00 g), alcohol (2:1–5:1 molar ratio vs. acid) and IL (5–30 mol% vs. acid) were placed in the reactor and the following conditions were applied: temperature between 40–80 °C for 60–240 min, stirring at 1000 rpm. The studies were carried out using different ILs (IL 3.0; IL 2.7 and IL 2.0). After the reaction mixture was homogenized with 4 mL acetonitrile and 0.3 g of *n*-decane was added as the internal standard, 40 μL of the solution was withdrawn, dissolved in 1.5 mL of acetonitrile and analysed using GC-FID. Yield and conversion were calculated based on the calibration curve prepared for the main product. The experiments were performed in triplicate and the errors were calculated as standard deviations.

### 2.5. Recycling Study

A recycling study was performed to confirm stability and reusability of ILs in the synthesis of both DEHS and DEHA. The reaction was carried out in a round-bottom reactor (25 mL), equipped with a reflux condenser, a temperature probe, an oil bath and a magnetic stirrer. Alcohol (butan-1-ol and 2-ethylhexan-1-ol; 4 mol), acid (succinic acid and adipic acid; 1 mol) and IL 2.7 (15 mol%) were placed in a reactor and the reaction was carried out at 80 °C, 17 Hz with reaction time of 240 min. After the reaction, the IL was isolated via phase separation, washed with water, dried on the rotary evaporator (80 °C, 1–2 mbar, 720 min) and used for another cycle of reaction. The upper phase was dissolved in 4 mL of acetonitrile, 0.3 g of *n*-decane was added as the internal standard and then 40 μL of that solution was withdrawn, dissolved in 1.5 mL of acetonitrile and analysed on GC-FID. Yield and conversion were calculated based on the calibration curve prepared for the main product. GC samples were analysed in triplicate and the errors were calculated as standard deviations.

## 3. Results

In preliminary studies, the suitable structure of ILs for the synthesis of dialkyl succinates and adipates was selected. Low-cost triethylamine and H_2_SO_4_ were chosen for the studies. Brønsted acidic ILs for the indicial tests were synthesised as described in the experimental section in the following molar ratios: 1:2.0, 1:2.7 and 1:3.0. Water was added to the reaction mixture to enable heat removal from the exothermic acid–base reaction [31,32]. After drying, the obtained ILs (IL 2.0; 2.7 and 3.0) were colourless liquids.

**Scheme 1:**
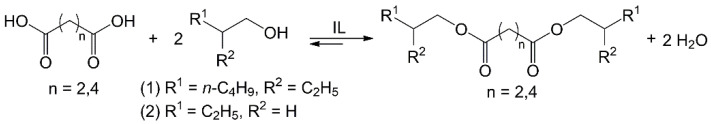
The esterification of succinic and adipic acids with butan-1-ol and 2-ethylhexan-1-ol.

**Scheme 2:**
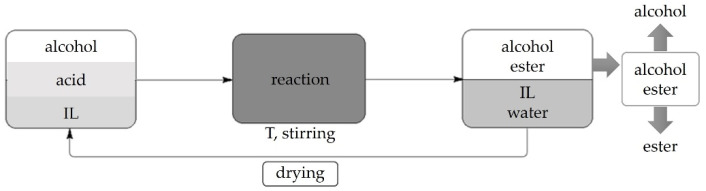
Overview of the esterification process adapted in this work.

**Figure 1:**
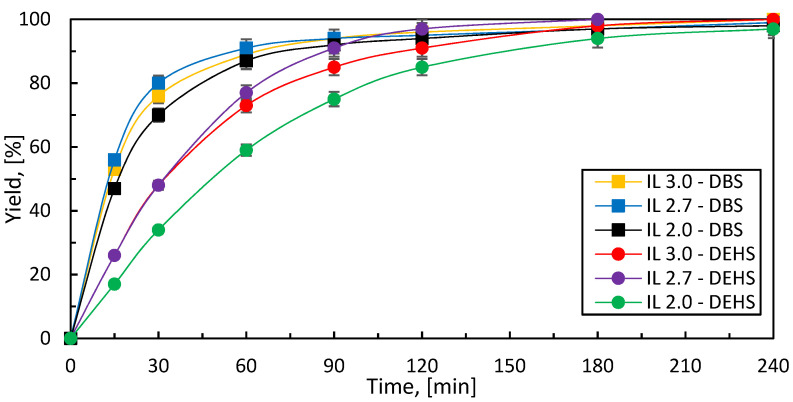
The influence of the IL composition on the esterification of succinic acid with butan-1-ol and 2-etylhexan-1-ol. Reaction conditions: alcohol 4 mol; succinic acid 1 mol; IL 2.7 (15 mol%/mol succinic acid (0.0025 mol)); 70 °C; 17 Hz.

Next, ILs 2.0, 2.7 and 3.0 (15 mol%/mol succinic acid) were used for the model esterification of succinic acid and butan-1-ol as well as 2-ethylhexan-1-ol (molar ratio 1:4) at 70 °C (Figure 1; Scheme 1). The reaction progress was monitored with GC-FID. In each case the full conversion of acid and 99 ± 2% of selectivity was observed with no traces of monoalkylated ester. During the reaction course, the formation of the second ester phase was observed and this facilitates the reaction progress, shifting the equilibrium towards the ester formation (Scheme 2).

In the first step, alcohol, acid and ILs are placed in the reactor. Alcohol and acid are partially miscible with IL. The increased temperature, vigorous mixing and ester/IL phase separation drives the esterification reaction. After completion of the reaction, two phases are separated. The IL phase after drying yields pure ILs that can be used in the next process. The upper layer is further processed to obtain the final ester. Distilled alcohol can be reused.

The obtained results showed that all three ILs were almost equally active for the synthesis of DBS; only slight differences in the reaction courses were observed for di(2-ethylhexyl) succinate (DEHS). All the studied ILs are characterised with a good thermal stability (*T*_d_ > 200 °C), which makes them suitable for this reaction [28]. Based on the respective commodity prices of triethylamine and sulphuric acid, if more sulfuric acid is introduced to the structure of the IL then it will be less expensive. Additionally, all of the studied ILs are characterised with a good thermal stability (*T*_d_ > 200 °C), which makes them suitable for this reaction [28]. Therefore, based on the ILs’ prices, the IL 3.0 would be the least expensive among the three investigated. However, it was observed that IL 3.0 changes its colour while exposed to 70 °C for several hours, turning slightly brownish, consequently affecting the colour of the final product. This behaviour was not observed for the IL 2.7, which appeared more heat resistant. Another important factor for consideration is the phase separation after the reaction. When IL 2.0 was used, the reaction phases were separated but the pH of the upper ester phase was slightly acidic, implying that traces of sulfuric acid or ILs were trapped there. On the other hand, IL 2.7 caused sharp phase separation and the pH of the ester layer was almost neutral. Based on this information, the most convenient IL for esterification of succinic acid is IL 2.7, which is hydrophilic enough for efficient phase separation, acidic enough to achieve high activity and stable during mixing at 70 °C.

**Figure 2:**
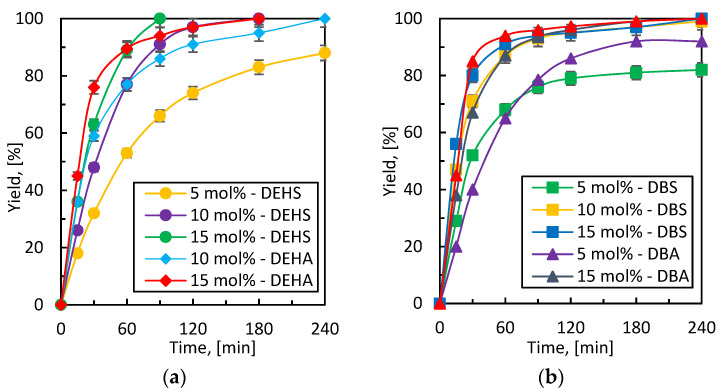
The influence of the amount of IL 2.7 on the on the esterification of succinic and adipic acid with (**a**) butan-1-ol and (**b**) 2-ethylhexan-1-ol. Reaction conditions: alcohol 4 mol; acid 1 mol; 70 °C (DBS, DEHS); 80 °C (DBA, DEHA); 17 Hz.

In the next step, the influence of the amount of IL 2.7, ranging from 5 to 30 mol%/mol acid, was studied for the synthesis of DBS, DEHS, di-n-butyl adipate DBA and di(2-ethylhexyl) adipate DEHA (Figure 2). The results showed that 5 mol% of IL was insufficient to overcome the equilibrium of esterification and the ester yields were low, about 80 ± 3%. Higher amounts of IL (above 10 mol%) are sufficient to obtain almost stochiometric amounts of esters, after the adequate time (90–240 min). Reaction times are generally shorter for di(2-ethylhexyl) esters due to the higher lipophilicity of the resulting esters and, as a consequence, lower phase mixability. As a result of these studies, 15 mol% was selected as the optimum amount of IL for the further experiments.

**Figure 3:**
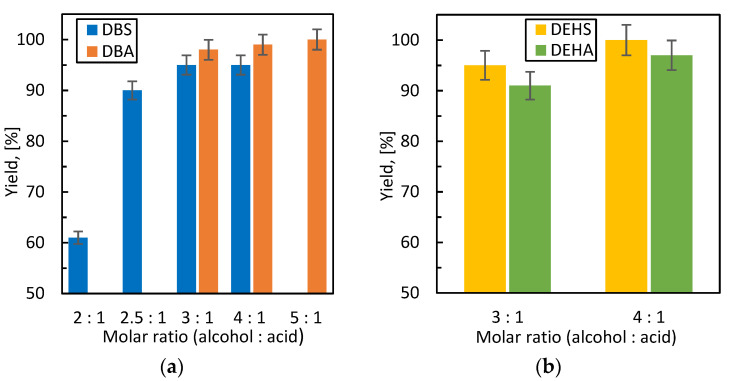
The influence of the amount of alcohol on the on the esterification of succinic and adipic acid with (**a**) butan-1-ol and (**b**) 2-ethylhexan-1-ol. Reaction conditions: acid 1 mol; IL 2.7 (15 mol%/mol succinic acid (0.0025 mol)); 60 °C (DBS, DEHS); 80 °C (DBA, DEHA); 17 Hz; reaction time for (**a**) 240 min, for (**b**) 120 min.

An important parameter, which can influence the esterification reaction yield, is the proportion of alcohol. To test how this will affect the reaction, excesses of 2 to 5 mol of alcohol per 1 mol of acid were studied (Figure 3). The results demonstrated that when an increasing excess of alcohol was used, higher yields were found, but only to a point. This is clearly visible with DBS (blue bars, Figure 3a), where a 2:1 ratio allowed us to obtain a yield of only 60 ± 2%. When a 3:1 ratio was applied, this yield increased to 95 ± 2% but did not change much with a further increase of the ratio to 4:1. Similar results were obtained for the other studied esters (Figure 3b).

**Figure 4:**
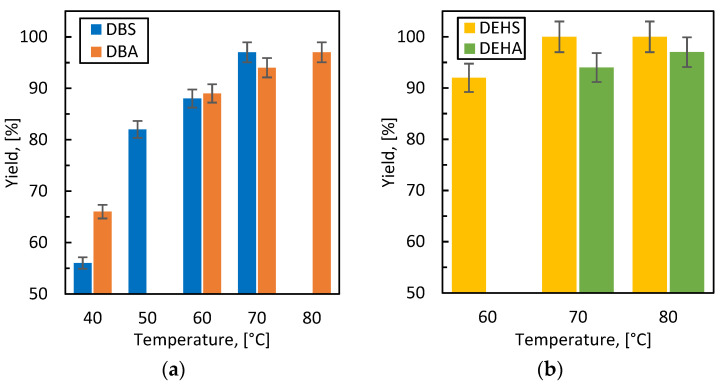
The influence of the temperature on the on the esterification of succinic and adipic acid with (**a**) butan-1-ol and (**b**) 2-ethylhexan-1-ol. Reaction conditions: alcohol 4 mol; acid 1 mol; IL 2.7 (15 mol%/mol succinic acid (0.0025 mol)); 17 Hz; reaction time 120 min.

The influence of temperature was studied to determine the yield of esterification after 120 min. A series of experiments was performed at 40, 50, 60, 70 and 80 °C. As expected, the ester yield increased with the temperature (Figure 4). Based on these results it can be concluded that it is favourable to carry out the reaction at 70 °C for the dialkyl succinates and at 80 °C for dialkyl adipates.

**Figure 5:**
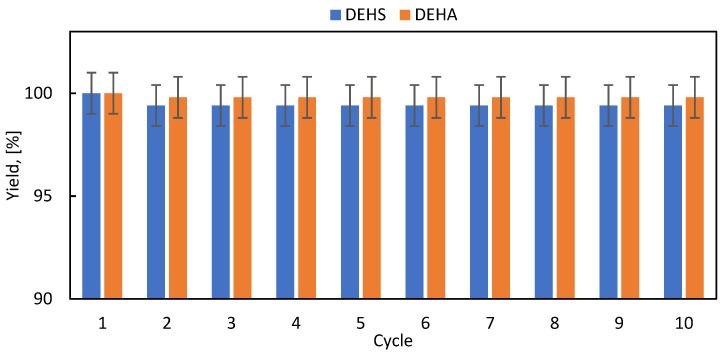
Recycle studies. Reaction conditions: IL 2.7 (15 mol%/mol succinic acid (0.0025 mol)); alcohol 4 mol; acid 1 mol; 80 °C (DEHS); 80 °C (DEHA), 17 Hz; reaction time 240 min.

Another important parameter for industrial application is the stability of the catalyst and the possibility of its further reuse in following processes. In order to test stability, a recycle study was performed. IL 2.7 was tested in ten (10) consecutive cycles for the synthesis of DEHS (blue bars, Figure 5) and DEHA (orange bars, Figure 5). After each cycle, the post reaction two-phase mixture was separated and the lower phase (containing mainly IL) was dried using rotary evaporator and used for the next cycle. The percent recovery of IL was high (99%), while the colour of ILs did not visibly change after 10 cycles. The ester-containing upper layer was dissolved in acetonitrile and analysed using GC-FID. As shown in Figure 5, the IL was stable and active in ten reaction cycles, measured by the yield (99 ± 1%) and selectivity (99 ± 1%) of the products.

After each cycle, the post reaction two-phase mixture was separated, the lower phase (mainly containing IL) was dried on the rotary evaporator and used for the next cycle. The upper layer containing ester was dissolved in acetonitrile and analysed using GC-FID. GC samples were analysed in triplicate and the errors were calculated as standard deviations.

## 4. Conclusions

In the pursuit of an effective method which is sustainable and combines the aspects of green chemistry with economic efficiency, we have studied the esterification of succinic and adipic acid with the alcohols butan-1-ol and 2-ethylhexan-1-ol in the presence of acidic IL. The IL was formed via a simple mixing of inexpensive triethylamine and sulphuric acid(V) with a small addition of water.

Currently, the problem of the development of alternative plasticizers is particularly weighty. The elaborated and cross-verified method overcomes some issues for the synthesis of plasticizers based on the dicarboxylic acids. The advantage of the described method is founded on the application of mild reaction conditions: 70–80 °C, using molar ratio of alcohol to acid 4:1 and only 15 mol% of catalyst. Moreover, the isolation of the product via a simple phase separation and alcohol distillation provides high purity of the final ester (>99%). The separated IL phase, after drying, can be recycled more than 10 times without any sacrifice in activity. It is worth emphasizing that the presence of monoesters has been not detected. These characteristics make this method extensively useful for industrial practice in the future.

The innovative method for the synthesis of dialkyl succinates and adipates presented in the article allows for solutions to some problems resulting from the use of conventional acids in the industrial production of these substances, such as toxicity, high temperatures and harmful waste. Additionally, the ionic liquids used in the tests are characterized by high acidity, high catalytic activity and stability under reaction conditions and their application potential underlines the simplicity of synthesis, easy separation from the reaction mixture, low price and the possibility of recycling to the next reaction cycle.

## 5. Patents

The following patent is the result of the described in this work studies: Chrobok, A.; Erfurt, K.; Latos, P.; Serwata, N.; Grymel, A.; Siwik, B.; Potajczuk-Czajka, K.; Grzybek, R. Process for preparing dialkyl succinates, From Pol. 2020, PL 234515 B1 20200331.
